# Progress Toward the Elimination of Mother-to-Child Transmission of Hepatitis B Virus — Worldwide, 2016–2021

**DOI:** 10.15585/mmwr.mm7130a2

**Published:** 2022-07-29

**Authors:** Nino Khetsuriani, Olufunmilayo Lesi, Shalini Desai, Paige A. Armstrong, Rania A. Tohme

**Affiliations:** ^1^Global Immunization Division, Center for Global Health, CDC; ^2^Global HIV, Hepatitis and STI Programmes, World Health Organization, Geneva, Switzerland; ^3^Immunization, Vaccines and Biologics Department, World Health Organization, Geneva, Switzerland; ^4^Division of Viral Hepatitis, National Center for HIV, Viral Hepatitis, STD, & TB Prevention, CDC.

Mother-to-child transmission (MTCT) of hepatitis B virus (HBV) often results in chronic HBV infection, the leading cause of cirrhosis and liver cancer (*1*). If not vaccinated, nine in 10 children infected at birth will become chronically infected. Globally, an estimated 6.4 million (range = 4.4–10.8 million) children aged ≤5 years are living with chronic HBV infection (*2*). In 2016, the World Health Assembly endorsed the goal to eliminate viral hepatitis as a public health threat by 2030, including the elimination of MTCT of HBV (*3*). Elimination of MTCT of HBV can be validated by demonstrating ≤0.1% prevalence of HBV surface antigen (HBsAg) among children aged ≤5 years, as well as ≥90% coverage with hepatitis B birth dose (HepB-BD) and 3 doses of hepatitis B vaccine (HepB3) (*4*,*5*). This report describes global progress toward elimination of MTCT of HBV during 2016–2021. By December 2020, 190 (98%) of 194 World Health Organization (WHO) member states* had introduced universal infant vaccination with hepatitis B vaccine (HepB), and 110 (57%) countries provided HepB-BD to all newborns. During 2016–2020, global HepB3 coverage remained between 82% and 85%, whereas HepB-BD coverage increased from 37% to 43%. In 2020, among the 99 countries reporting both HepB3 and HepB-BD coverage, 41 (41%) achieved ≥90% coverage with both. By December 2021, serosurveys documented ≤0.1% HBsAg prevalence among children in 11 countries. Accelerating HepB-BD introduction, increasing HepB3 coverage, and monitoring programmatic and impact indicators are essential for elimination of MTCT of HBV.

## Immunization Activities

Because immunization is a key intervention to prevent MTCT of HBV, WHO recommends that all newborns receive a timely HepB-BD^†^ dose followed by 2–3 additional HepB doses, according to national schedules (*1*). Countries report immunization data to WHO annually through the WHO and UNICEF Joint Reporting Form. WHO and UNICEF review reported coverage data and surveys to generate country-specific coverage estimates.^§^ This activity was reviewed by CDC and was conducted consistent with applicable federal law and CDC policy.^¶^

By 2020, 190 (98%) of 194 countries had introduced universal infant hepatitis B vaccination compared with 186 (96%) in 2016. In 2020, 110 (57%) countries provided HepB-BD** to all newborns, a 10% increase from 100 (52%) in 2016. During 2016–2020, 33 to 34 (17%–18%) countries, mostly in the European Region, administered HepB-BD selectively to newborns of HBsAg-positive mothers (i.e., selective or targeted birth dose vaccination) each year.^††^ The number of countries that had not introduced routine HepB-BD vaccination declined by 15%, from 60 (31%) in 2016 to 51 (26%) in 2020^§§^ (Table 1). Most of these countries are in the African Region where 34 (72%) of 47 countries do not provide a HepB-BD.

**TABLE 1:** Hepatitis B vaccination policies and coverage with ≥3 doses of hepatitis B vaccine and with hepatitis B vaccine birth dose — worldwide, 2016–2020*

Variable	Countries, No. (%)				
	2016	2017	2018	2019	2020
**HepB vaccination policy^†,§^**					
Universal, infant	186 (96)	188 (97)	189 (97)	190 (98)	190 (98)
Universal, children aged ≥1 yr	3 (2)	3 (2)	2 (1)	1 (1)	1 (1)
Selective	5 (3)	3 (2)	3 (2)	3 (2)	3 (2)
**HepB-BD vaccination policy^†^**					
Universal	100 (52)	104 (54)	106 (55)	109 (56)	110 (57)
Selective	34 (17)	34 (18)	34 (17)	33 (17)	33 (17)
HepB-BD not introduced	60 (31)	56 (29)	54 (28)	52 (27)	51 (26)
**Immunization coverage**					
HepB3 coverage reported^¶^	185 (99)	185 (98)	186 (98)	189 (99)	189 (99)
HepB3 coverage ≥90%**	116 (63)	113 (61)	116 (62)	119 (63)	98 (52)
Timely HepB-BD coverage^††^ reported^§§^	80 (80)	90 (87)	92 (87)	96 (88)	99 (90)
Timely HepB-BD coverage ≥90%^¶¶^	46 (58)	46 (51)	50 (54)	53 (55)	53 (54)
Both HepB3 and HepB-BD coverage reported^†^	80 (41)	90 (46)	92 (47)	96 (49)	99 (51)
Both HepB3 and HepB-BD coverage ≥90%***	43 (54)	42 (47)	46 (50)	51 (53)	41 (41)
**HepB3 coverage, global and regional, %^†††^**					
Global	84	84	84	85	82
**Regions**					
African	73	74	74	75	73
Americas	88	84	83	79	81
Eastern Mediterranean	81	83	84	85	81
European	82	84	85	92	91
South-East Asia	89	90	90	91	86
Western Pacific	93	92	90	94	94
**Timely HepB-BD coverage, global and regional, %^†††^**					
Global	37	42	42	44	43
**Regions^†^**					
African	10	10	12	15	16
Americas	50	54	56	55	60
Eastern Mediterranean	20	33	33	33	33
European^§§§^	41	41	42	44	43
South-East Asia	34	45	48	53	51
Western Pacific	83	84	83	84	81

**Abbreviations:** HepB = hepatitis B vaccine; HepB3 = third dose of HepB; HepB-BD = birth dose of HepB; WHO = World Health Organization.

* https://immunizationdata.who.int/pages/coverage/hepb.html?CODE=Global&GROUP=WHO%20Regions+Countries&ANTIGEN=&YEAR=

^†^ Among all 194 WHO member states. https://www.who.int/countries

^§^ HepB vaccination policy: universal = all persons in the applicable age group (i.e., all infants, children aged 1–12 years, or adolescents aged 13–15 years for routine HepB vaccination, and all newborns for HepB-BD) receive HepB; selective = only infants born to mothers with positive HBsAg test results receive HepB vaccination, starting with HepB-BD.

^¶^ Among countries with universal infant HepB vaccination policy.

** Among countries that reported HepB3 coverage.

^††^ Timely HepB-BD is defined as a dose of HepB given within 24 hours of birth.

^§§^ Among countries with universal HepB-BD policy.

^¶¶^ Among countries that reported HepB-BD coverage.

*** Among countries that reported both HepB3 and HepB-BD coverage.

^†††^ Global or regional coverage = a weighted sum of WHO/UNICEF estimates of national coverage (WUENIC) by target population from the United Nations Population Division’s World Population Prospects.

^§§§^ For all countries in the European region, including 30 countries with selective HepB-BD policies that do not report HepB-BD coverage to WHO. This results in lower regional estimate than the actual coverage in countries with universal HepB-BD policies that report this information to WHO.

During 2016–2020, global coverage with HepB3 remained between 82% and 85%, whereas timely coverage with HepB-BD increased from 37% to 43%. During this period, regional HepB3 and HepB-BD coverages were highest in the Western Pacific Region and lowest in the African Region (Table 1). During 2016–2019, HepB3 coverage was ≥90% in 61%–63% of reporting countries; this proportion declined to 52% in 2020. HepB-BD coverage was ≥90% in 51%–58% of reporting countries, with the highest proportion (58%) observed in 2016 and lowest (51%) in 2017. During 2016–2019, among countries that reported coverage with HepB3 and HepB-BD, 47%–54% reported ≥90% coverage for both; this proportion declined to 41% in 2020 (Table 1).

## Other Interventions to Prevent Mother-to-Child Transmission

To prevent MTCT of HBV, countries with selective HepB-BD vaccination policies rely on antenatal screening combined with antiviral treatment for eligible HBsAg-positive pregnant women and postexposure prophylaxis for HBV-exposed infants^¶¶^ (*1*). Information on the performance of these interventions is usually not reportable and is collected through special studies.

In 2020, among 33 countries with selective HepB-BD vaccination policies, 32 (97%) implemented nationwide antenatal hepatitis B screening, with ≥90% coverage in 17 (89%) of 19 countries with available information. HepB-BD coverage among infants born to HBV-infected mothers was ≥90% in all nine countries with available information (*6*–*8*).

## HBsAg Seroprevalence in Children and Mother-to-Child Transmission Rate

For countries with a universal HepB-BD vaccination policy, the impact target to achieve elimination of MTCT of HBV is ≤0.1% HBsAg prevalence among children aged ≤5 years; for countries with a selective HepB-BD policy, the impact target also includes an MTCT rate ≤2% (Table 2) (*4*,*5*). In 2019, WHO estimated global HBsAg prevalence among children aged ≤5 years to be 0.9%, with prevalence ranging from 0.1% in the Region of the Americas to 2.5% in the African region (Table 3) (*2*). According to a modeling study, HBsAg prevalence among children aged 5 years in 2016 was ≤0.1% in 52 of 119 countries assessed (*9*); by December 2021, 11 countries*** had demonstrated HBsAg prevalence ≤0.1% in representative serosurveys. Studies in two countries^†††^ with selective HepB-BD demonstrated an MTCT rate ≤2% (Table 3).

**TABLE 2:** Impact and programmatic targets for validation of elimination of mother-to-child transmission of hepatitis B — World Health Organization, 2021*

Target	Description
**Countries with universal HepB-BD vaccination policy†**	
**Impact target**	
≤0.1% HBsAg prevalence in children aged ≤5 yrs	Childhood HBsAg prevalence is a proxy for HBV incidence. Reflects cumulative incidence from perinatal and early horizontal transmission. Preferably measured in representative serosurveys among children aged ≤5 yrs. For regions and countries with a long history of high hepatitis B vaccination coverage, serosurveys conducted in children aged >5 yrs (e.g., school-based surveys), can be acceptable. If implementing a serosurvey is not feasible, a mathematical modeling of the impact indicator based on available representative empirical data may be considered. Triangulation of methods is recommended.
**Programmatic targets^§^**	
≥90% HepB3 national infant immunization coverage	National coverage with ≥3 doses of hepatitis B vaccine.
≥90% timely HepB-BD national immunization coverage	National coverage with timely HepB-BD; timely HepB-BD is defined as a dose of HepB given within 24 hrs of birth.
**Additional programmatic target**	
≥80% HepB3 and HepB-BD coverage in all provinces or subnational areas	To provide supportive evidence for equity consideration; not required for validation. Demonstrates lack of heterogeneity in coverage throughout the country.
**Countries with selective HepB-BD vaccination policy^¶^**	
**Impact target**	
≤0.1% HBsAg prevalence in children aged ≤5 yrs	Same as for countries with universal HepB-BD.
**Additional impact target**	
≤2% MTCT rate	MTCT rate measures the proportion of HBsAg-positive infants among HBV-exposed infants (i.e., those born to HBsAg-positive mothers). Infant’s HBV infection status is determined based on the results of post-vaccination serology testing of exposed infants aged 9–12 mos.
**Programmatic targets^§^**	
≥90% HepB3 national infant immunization coverage	Same as for countries with universal HepB-BD.
≥90% timely HepB-BD immunization coverage among HBV-exposed infants**	
≥90% coverage with hepatitis B antenatal screening	Percentage of pregnant women in antenatal care tested for hepatitis B.
≥90% coverage of eligible HBsAg-positive pregnant women with antiviral treatment against HBV	Eligibility is determined in accordance with national policies or WHO guidance on use of antiviral prophylaxis for prevention of MTCT of HBV.

**Abbreviations:** HBsAg = hepatitis B virus surface antigen; HBV = hepatitis B virus; HepB3 = third dose of hepatitis B vaccine; HepB-BD = birth dose of hepatitis B vaccine; MTCT = mother-to-child transmission; WHO = World Health Organization.

* https://www.who.int/publications/i/item/9789240028395 and https://www.who.int/publications/i/item/9789240039360

^†^ Countries with universal HepB-BD vaccination policy administer HepB-BD to all newborns.

^§^ All programmatic targets must be achieved and maintained for at least 2 years.

^¶^ Countries with selective HepB-BD vaccination policy administer HepB-BD to hepatitis B-exposed newborns only.

** HBV-exposed is defined as born to an HBsAg-positive mother.

**TABLE 3:** Estimated and directly measured hepatitis B virus surface antigen seroprevalence and mother-to-child transmission rate, by World Health Organization region — select countries, worldwide, 2008–2021

Variable	Prevalence, (range), %
WHO modeling estimates*	
**HBsAg seroprevalence among children aged <5 yrs for 2019**	
Globally	0.9 (0.7–1.6)
**Regions^†^**	
African	2.5 (1.7–4.0)
Americas	0.1 (<0.1–0.2)
Eastern Mediterranean	0.8 (0.5–1.1)
European	0.3 (0.1–0.5)
South-East Asia	0.4 (0.3–1.0)
Western Pacific	0.3 (0.2–0.5)
**Direct measurements^§^**	
**HBsAg seroprevalence among children (yrs)**	
Bangladesh (2011–2012)^¶^	0.05 (0.0–0.1)
Brunei (2011)**	0.1 (NR)
Colombia (2019)^††^	0.0 (0.0–0.09)
Cook Islands (2012)**	0.0 (NR)
Fiji (2008)**	0.0 (NR)
Georgia (2021)^§§^	0.03 (0.0–0.19)
Niue (2015)**	0.0 (NR)
Palau (2008)**	0.0 (NR)
Samoa (2014)**	0.09 (NR)
Spain (2015)^¶¶^	0.0 (NR)
Thailand (2014)***	0.1 (NR)
**HBV MTCT rate,^†††^ % (yrs)**	
Japan (2014–2016)^§§§^	2.0
United Kingdom (2014–2019)^¶¶¶^	<0.5

**Abbreviations:** HBsAg = Hepatitis B virus surface antigen; HBV = hepatitis B virus; MTCT = mother-to-child transmission; NR = not reported; WHO = World Health Organization.

* https://doi.org/10.1016/s2468-1253(18)30056-6

^†^ https://www.who.int/countries

^§^ Methodologies for seroprevalence and MTCT rate data sources: disease modeling (WHO estimates), representative population-based serosurveys (Bangladesh, Brunei, Fiji, Georgia, Palau, Samoa, Spain, and Thailand), census surveys (Cook Islands and Niue), two-phase classification survey (Colombia), national survey of antenatal screening sites (Japan), analysis of routinely collected antenatal screening program data (United Kingdom).

^¶^ https://www.ajtmh.org/view/journals/tpmd/99/3/article-p764.xml

** https://www.who.int/publications/i/item/9789290616986

^††^ https://doi.org/10.1111/jvh.13719

^§§^ https://ncdc.ge/#/pages/file/b08a70c2-44a1-4279-9d3b-6145dd98ea51

^¶¶^ https://doi.org/10.15585/mmwr.mm7030a1

*** https://doi.org/10.1371/journal.pone.0150499

^†††^ HBV MTCT rate is the percentage of infants with chronic HBV infection among infants born to HBsAg-positive mothers.

^§§§^ https://doi.org/10.1002/ygh2.441

^¶¶¶^ National data submitted to the European Regional Hepatitis B Working Group, 2022.

## Validation

The Global Validation Advisory Committee for elimination of MTCT of HIV and syphilis was established in 2015. In 2021, the Committee’s role was expanded to include validation of elimination of MTCT of HBV. WHO revised the global guidance on the validation of elimination of MTCT to include “triple” elimination of HIV, syphilis, and hepatitis B (*5*). The programmatic and impact indicators for validation of elimination of MTCT of HBV vary according to countries’ HepB vaccination programs (Table 2).

Piloting of validation instruments in seven countries^§§§^ demonstrated feasibility of their use. Representative serosurvey data to support direct impact measurement were available in five countries.^¶¶¶^ In England (the pilot did not include the rest of the United Kingdom), HBsAg prevalence and the MTCT rate were extrapolated from routinely collected antenatal screening data. National HepB immunization coverage data were available in all seven pilot countries; subnational data were available in five.****

## Discussion

Substantial progress has been made toward elimination of MTCT of HBV in most WHO regions. Globally, 41 countries reported ≥90% coverage with both HepB-BD and HepB3, a critical component of elimination of viral hepatitis as a public health problem by 2030. Successful implementation of HepB vaccination and other interventions to prevent MTCT globally resulted in a substantial decrease in HBV prevalence among children in all regions except for the African region (*2*).

Currently, nearly all countries include HepB in their routine infant immunization schedules; however, during 2016–2020, little change in global coverage for HepB3 and HepB-BD was observed. The introduction of HepB-BD into routine immunization programs in 10 additional countries during 2016–2020 is encouraging. However, the slow increase in the number of countries that include HepB-BD in their routine immunization programs suggests that this process has stalled, especially in the African Region. Further, service disruptions caused by the COVID-19 pandemic contributed to the decline of the immunization coverage with HepB in 2020, particularly for HepB3 (*10*). To meet programmatic targets for elimination of MTCT of HBV, interventions to mitigate the pandemic’s impact on immunization systems need to be implemented (*10*).

Accelerating the introduction of HepB-BD into the routine immunization programs of remaining countries is essential for achieving global elimination of MTCT of HBV. The African region, which has a high prevalence of chronic HBV infection (*2*) and where HepB-BD introduction is lagging, requires special attention. Increasing demand among pregnant women and awareness among policymakers and health care workers, improving links between maternal and child health and immunization programs, and ensuring sustainable support would help with successful implementation of HepB-BD vaccination.

Data on impact measures to support validation of elimination of MTCT of HBV are currently available for only a few countries. Countries that have met immunization coverage targets are encouraged to conduct serosurveys to document HBsAg prevalence. Implementing nationwide hepatitis B serosurveys is challenging, given a large sample size and considerable resource requirements. Integration with other serosurveys^††††^ or use of multiphase methodology surveys^§§§§^ could help reduce implementation costs. Although mathematical modeling is not a substitute for serosurveys, triangulation of various data sources could be considered in assessing the elimination of MTCT of HBV.

To better assess progress toward meeting the elimination targets, countries with selective HepB-BD will need to establish data systems to document performance measures of additional interventions to prevent MTCT of HBV^¶¶¶¶^ (*4*,*5*). Most HBsAg-positive mothers in countries with historically low HBV prevalence come from countries where prevalence is high (*6*); therefore, ensuring equal access for foreign-born women to antenatal services and MTCT prevention interventions is important.

The findings in this report are subject to at least two limitations. First, missing immunization data from some countries that did not report to WHO prevent accurate assessment of global and regional coverage. Second, in countries with selective HepB-BD vaccination, limited data availability hampers evaluation of their progress toward elimination of MTCT.

Elimination of MTCT of HBV is achievable with the currently available tools; based on modeled estimates, Elimination of MTCT might have already been attained in several countries (*9*). Countries will be able to apply for validation once the standardized tools are finalized. For countries with a high prevalence of HBV that do not yet have the capability to achieve impact targets, milestones known as the Path to Elimination which assess progress toward achieving programmatic targets (*5*) are available to measure progress toward elimination of MTCT. Integration of activities to prevent MTCT of HBV with interventions to prevent MTCT of HIV and syphilis provides the opportunity to synergize across these programs to help achieve triple elimination. Once achieved globally, elimination of MTCT of HBV will result in removing perinatal transmission as a source of chronic HBV infections and will be an important milestone toward achieving elimination of viral hepatitis as a public health threat.

**Summary**

**What is already known about this topic?**

Mother-to-child transmission of hepatitis B virus (HBV), a leading cause of liver cancer, is targeted for global elimination.

**What is added by this report?**

During 2016–2020, global coverage with the third dose of hepatitis B vaccine remained between 82% and 85%, whereas timely coverage with hepatitis B birth dose increased from 37% to 43%. Coverage in 2020 was ≥90% for both the hepatitis B birth dose and the 3-dose series of hepatitis B vaccine in 41% of countries. In 11 countries, prevalence of HBV surface antigen among children was ≤0.1%.

**What are the implications for public health practice?**

Accelerating hepatitis B birth dose introduction, increasing coverage with the third dose of hepatitis B vaccine, and monitoring programmatic and impact indicators are essential for elimination of mother-to-child transmission of HBV.

## Footnotes

*
https://www.who.int/countries

† Timely HepB-BD is defined as a hepatitis B vaccine dose administered within 24 hours of birth.

§ Most recent available WHO/UNICEF estimates of national immunization coverage were for 2020. https://immunizationdata.who.int/pages/coverage/hepb.html?CODE=Global&GROUP=WHO%20Regions+Countries&ANTIGEN=&YEAR=

¶ 45 C.F.R. part 46, 21 C.F.R. part 56; 42 U.S.C. Sect. 241(d); 5 U.S.C. Sect. 552a; 44 U.S.C. Sect. 3501 et seq.

** Referred to as universal HepB-BD vaccination.

†† Thirty (91%) of 33 countries implementing selective HepB-BD in 2020 were in the European Region, two (6%) were in the Western Pacific Region, and one (3%) was in the Region of the Americas.

§§ In 2020, 34 (67%) of 51 countries that had not yet introduced HepB-BD were in the African Region, nine (18%) in the Region of the Americas, five (10%) in the Eastern Mediterranean Region, and three (6%) in the South-East Asia Region.

¶¶ WHO recommends using the same treatment criteria for pregnant and nonpregnant persons: antiviral treatment against HBV for infected persons with HBV viral load >200,000 IU/mL (or, in the absence of DNA testing, for HBeAg-positive persons). Postexposure prophylaxis for HBV-exposed newborns (i.e., those born to HBsAg-positive women) includes administration of timely HepB-BD and 2 or 3 subsequent HepB doses, and where feasible, administration of hepatitis B immune globulin at birth. In addition, these infants may be offered postvaccination serology testing at age 9–12 months to determine their HBV infection status. https://apps.who.int/iris/bitstream/handle/10665/333391/9789240002708-eng.pdf?sequence=1&isAllowed=y

*** Brunei, Cook Islands, Fiji, Niue, Palau, and Samoa in the Western Pacific Region; Georgia and Spain in the European Region; Colombia in the Region of the Americas; and Bangladesh and Thailand in the South-East Asia Region.

††† Japan and the United Kingdom.

§§§ Brazil, Egypt, England, Georgia, Mongolia, Rwanda, and Thailand.

¶¶¶ Egypt, Georgia, Mongolia, Rwanda, and Thailand.

**** Brazil, England, Georgia, Mongolia, and Thailand.

††††
https://ncdc.ge/#/pages/file/b08a70c2-44a1-4279-9d3b-6145dd98ea51

§§§§
https://onlinelibrary.wiley.com/doi/10.1111/jvh.13719

¶¶¶¶ Including coverage with antenatal screening for HBsAg, antiviral treatment of eligible pregnant women, and HepB3 and HepB-BD coverage and post-vaccination serology testing of exposed infants.
